# Acute Otitis Media

**Published:** 1909-01-02

**Authors:** 


### Otology.

### ACUTE OTITIS MEDIA.

Acute inflammations of the middle ear practically always originate by the spread of inflammation through the Eustachian tube; the exciting cause is therefore some form of inflammatory disease in the nose or pharynx. They are predisposed to by any form of nasal obstruction, especially adenoids, although the latter need not necessarily be large enough to produce marked nasal obstruction. The affection is commonest in children and young adults; second attacks are quite common, and acute otitis in an elderly patient is usually but a recrudescence of former suppuration. A simple coryza is often the exciting cause, but the affection is especially common as a complication of the specific fevers, scarlet fever, measles, and influenza.

Acute otitis media is often classified into catarrhal and suppurative varieties, but they all begin in the same way, and it is better to say that resolution occurs in some cases without proceeding to suppuration. The symptoms are a sensation of stuffiness in the ear, deafness, tinnitus, pain, and fever. The temperature generally rises to about 102° at night, but rarely higher than 103° unless suppuration occurs, in which case there may be very severe constitutional disturbance with rigors, vomiting, and, in children, convulsions and symptoms of cerebral irritation. Suppuration and perforation of the drum may, however, take place without the appearance of very marked constitutional disturbance. Unless intracranial complications are present, the symptoms improve rapidly after perforation of the drum. Pain is severe and worse at night; it is located deep in the canal or beneath the tragus. If the pain be most marked behind the ear over the mastoid process, the condition is probably one of acute mastoiditis. The antrum is always involved in cases of acute otitis media, but if drainage into the tympanum is good, the inflammation subsides after perforation. Some tenderness over the antrum during the acute stage is not therefore an absolute indication for the mastoid operation, but becomes so if it persist after free incision or perforation of the drum.

In the earliest stage the membrana tympani is injected, the hyperæmia being most marked along the handle of the malleus; later it becomes uniformly red and dull, and the anatomical features are completely lost. If there be much exudation, bulging of the drum occurs, usually in its upper and posterior part; sometimes, when perforation is imminent a yellowish colouration is seen in the middle of the bulging area. Perforation generally occurs in the lower half of the membrane; the opening is often minute and difficult to see, and it frequently shows itself as a tiny pulsating point of light. Perforation occurs by definite destruction of the tissues, and is consequently far less likely to heal than an incision made by the surgeon.

It is important to emphasise the fact that permanent aural discharge and the serious and fatal complications which are so frequently encountered are eminently preventable in the very great majority of cases by correct treatment at an early stage. Otitis media can be made much less frequent and far less severe by proper attention to the nose and throat, more especially in children. This attention is a very simple matter. It consists in the removal of adenoids and other nasal abnormalities when they are causing symptoms of nasal obstruction or frequent catarrhs and, especially, transient deafness or earache; and the use of a mild antiseptic and alkaline lotion to the nares when rhinitis is present and during the course of the specific fevers. In adults and older children the lotion should be introduced warm by means of a small rubber ball-syringe; in young children it may be applied as a coarse spray, or simply dropped into the nostrils from an aural dropper or the filler of a stylographic pen.

When otitis is present it should be treated promptly by rest in bed, low diet and purgatives; poultices must never be used, but hot, dry flannels may be applied for the relief of pain. Guttæ containing opium may be employed, but a solution of carbolic acid in glycerine, gr. 20 to the ounce, is preferable, being a useful sedative and antiseptic. The application of four or six leeches to the mastoid process is of value. In the earliest stage, and in skilled hands, gentle inflation by the Eustachian catheter will sometimes give exit to the exudation and abort the attack; Politzer's bag should never be used. All these methods frequently fail to prevent suppuration, and it cannot be too strongly stated that the only treatment in cases proceeding to suppuration is incision of the drum. By this means pain is at once relieved, inflammation ceases to extend and resolution commences. The wound heals quickly without any destruction of the drum, and it is far better to perform paracentesis of the drum too soon than a minute too late. A bulging drum is an absolute indication for the operation, but it is often advisable to incise before bulging appears. It is not too much to say that a natural perforation of the drum should never be allowed to occur, and that if all cases of acute otitis were incised in time, chronic suppurations and intracranial complications would be almost unknown. The operation should be performed with strict antiseptic precautions, and the ear lightly plugged and dressed daily.

